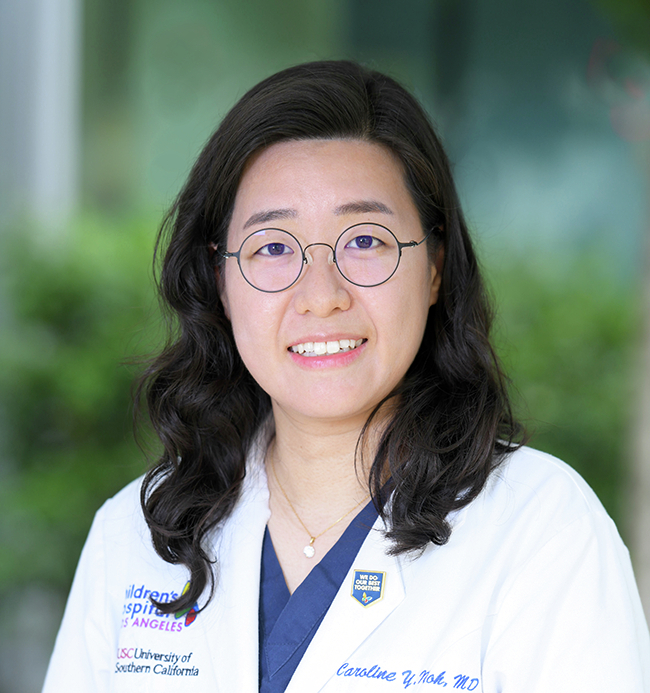# Caroline Y. Noh: Early Career Investigator biocommentary

**DOI:** 10.1038/s41390-025-04755-x

**Published:** 2026-01-17

**Authors:** Caroline Y. Noh

**Affiliations:** Division of Neonatology, Fetal and Neonatal Institute, Children’s Hospital Los Angeles, University of Southern California Keck School of Medicine, Los Angeles, CA USA

“What if premature infants didn’t have to struggle on life support, but could instead continue developing in an artificial womb?” This question blazed in my mind in 2008 in the classroom at Ajou University School of Medicine in South Korea, when I first learned about prematurity and extracorporeal membrane oxygenation (ECMO). I envisioned developing an extracorporeal placenta with amniotic fluid where premature infants could thrive. This aspiration ignited my passion for Neonatal-Perinatal Medicine.

After spending 6 months traveling across the United States as a visiting medical student—staying one month at each institution, eating instant ramen daily in youth hostels and budget rooms—to determine if I would enjoy building a life in the USA, I recognized the unparalleled training opportunities in the USA.

I completed a residency in Pediatrics at the University of Florida and Shands Children’s Hospital in Gainesville, FL, as the only international medical graduate in my class of 17—an experience that taught me resilience. I met Robert Lawrence and his mother, Ruth Lawrence, exemplary pediatricians whose lives and mentorship profoundly shaped my approach to clinical medicine. Additionally, Josef Neu introduced me to evidence-based neonatology and helped me develop the scholarly rigor necessary for clinical research.

At Stanford, where I pursued fellowship training in Neonatal-Perinatal Medicine and earned a master’s degree in Epidemiology and Clinical Research, I was blessed to train under Krisa Van Meurs, Valerie Chock, and Shazia Bhombal. Through their complementary expertise in congenital diaphragmatic hernia (CDH), neurohemodynamic monitoring, and neonatal hemodynamics, I had the privilege of receiving multifaceted mentorship to incorporate all aspects into my first CDH research projects, where our team investigated early echocardiographic predictors of mortality and ECMO use as well as the impact of inhaled nitric oxide (iNO) on these outcomes. Both led to publications in Pediatric Research, including the article featured in this issue.

As both a clinician and researcher, I am committed to advancing the field of CDH and neonatal hemodynamics, while working at Children’s Hospital Los Angeles and the University of Southern California, where I serve as Associate Director of Neonatal Hemodynamics and Point-of-Care Ultrasound Program and site PI for the NoNO trial, an NIH-funded multicenter randomized trial that investigates the impact of iNO in neonates with CDH.

What I learned and want to share with those beginning an academic career is to seek mentors who are genuinely invested in your growth and success. The people who champion you are as important as the work you do. Find your advocates; be someone else’s advocate; and don’t be afraid to step into opportunities that feel a little too big. That’s where the growth happens.